# Effects of recycled waste on the modulus of elasticity of structural concrete

**DOI:** 10.1038/s41598-024-65516-0

**Published:** 2024-07-13

**Authors:** Najib N. Gerges, Camille A. Issa, Nariman J. Khalil, Sarah Aintrazi

**Affiliations:** 1 Department of Civil Engineering, Lebanese American University, Byblos, Lebanon; 2 Department of Civil Engineering, University of Balamand, Balamand, Lebanon

**Keywords:** Modulus of elasticity, Recycled waste, Structural concrete, Engineering, Materials science

## Abstract

Concrete, the construction industry’s most utilized construction material, has transformed the environment and the modern built-up lifestyle. Although concrete is a first-rate supplier to the carbon footprint, it is imperative for buildings to display sustainable characteristics. Scholars have explored techniques to lessen the carbon footprint and the way to put into effect strategic waste control plans in which waste is reused. This study explores the dual benefits wherein concrete ingredients are replaced through abandoned waste which reduces the unwanted waste materials that have a substantial carbon footprint and thus results in the recycling of waste as part of a sustainable economic system. In this study, timber ash is utilized as a partial substitute for sand and cement, crumb rubber and waste glass as a partial substitute for sand, recycled concrete, and waste glass as a substitute for gravel. Characteristics studies were done to check the influence of each waste replacement on the modulus of elasticity of concrete. More than sixty-five combinations of waste have been examined to attain the modulus of elasticity of concrete. A total of about 200 concrete cylinders were cast to provide at least three cylinders for each generated data point. Three different ASTM standards were utilized to determine the modulus of elasticity of each mix. Four mixes comprising of the combination of two waste materials and two mixes comprising of the combination of three waste materials replacing natural materials were determined to exhibit an equal or superior modulus of elasticity of the control mix of 25 GPa.

## Introduction

The reduction of pollutants is a project for all international environmentally conscious entities, especially with the increasing emission of greenhouse gases into the environment and discharges from waste in uncontrolled landfills. Furthermore, zones existing for landfills are rare, waste consisting of used tires, waste glass, wood ash, construction and demolition waste is generated every day. Those wastes last in landfills for years because they are typically non-biodegradable. The combination of recycled waste in the manufacturing of concrete is reasonable and attractive for several purposes: lowering the consumption of natural materials, decreasing CO_2_ emissions into the surroundings due to aggregate extraction, and additionally improving the viability of the construction industry. It is estimated that the annual utilization of virgin aggregates in the concrete mix design is about ten billion tons^1^. According to the World Bank^2^, the world annually produces two billion tons of municipal nonbiodegradable waste, of which at least thirty percent is disposed of in an environmentally professional manner. As a result, the environment has been exposed to a tremendous amount of waste material. In addition, the buildup industry consumes a massive amount of virgin materials for the manufacturing of concrete^3^. The usage of recycled waste aggregates in concrete mix design offers an alternative environmentally friendly and cost effective route^4^. The mining of natural aggregates, the making of cement, and all the manufacturing processes associated with the making of concrete result in the emission of a tremendous amount of CO_2_ into the environment. Because of this, the incorporation of recycled waste in the production of concrete represents an extensive step in the direction of a greater sustainable society.

Landfilling is one of the most significant techniques for managing industrial and municipal waste, but it additionally contributes appreciably to ecological pollutants. Further to the probable risks that landfilling poses to groundwater and soil, it additionally triggers the release of poisonous orders and gases in case it is not professionally managed. Methane (CH_4_) and carbon dioxide (CO_2_) are among the significant greenhouse gases released from landfills^5^. Those discharged orders and gases have an adverse effect on the environmental welfare. The World Health Organization (WHO)^6^ estimates that approximately ninety-one percent of the planet population exists in places wherein air quality surpass desirable limits. The disposal of tires by way of incineration creates enormous amounts of CO_2_, benzene, and different poisonous materials due to their notably flammable tendencies. For instance, old tires take at a least a century to disintegrate naturally, causing soil poisoning and the release of chemical compounds, and almost one fourth of discarded tires are dumped in landfills yearly^7^. The recycling is the best and most powerful ecological manner of disposing of this waste. Discarded dumped tires can be grinded into crumb rubber (CR) and included into concrete mix designs^8^. Gerges et. al.^9^ concluded that the optimum percentage of CR replacing sand in the concrete mixture is five percent to keep maximum compressive strength and ten percent to keep the maximum flexural capacity. Huang et al. al^10^ predicted an experimental model linking the CR content to concrete compressive strength. Wang and Du^11^ empirically established that sound, thermal insulation, and anti-noise benefits is resulted from incorporating CR into the concrete mix.

Glass is another non-biodegradable yet a hundred percent reusable waste dumped in landfills. It hypothetically takes a bottle a million years to decompose within the ecosystem, or still slower in case it exists in a landfill. The US Environmental Protection Agency (EPA), indicated that glass accounts for nearly five percent of the yearly landfilled municipal solid, which comprises of six million tons of glass yearly^12^. The addition of waste glass in concrete mix design is feasible due to the similarity of the chemical and physical properties of glass and sand^13^. Recently, several papers^14–16^ have highlighted the advantages and limitations of combining waste glass in concrete.

Construction and demolition waste (CDW) which are debris produced in the process of the renovation, rehabilitation and demolition of bridges, buildings, and concrete road pavements are mostly dumped in landfills. The EPA promotes and identifies CDW as sources that might be utilized in new construction projects, eliminating the requirement to excavate and procedure virgin materials^17^. In 2018, the United States accumulated six hundred million tons of CDW, that is greater than double the quantity of municipal nonbiodegradable waste produced, and is generally dumped in landfills with exhaustible volumetric results. Novková and Mikulić^18^, indicated that recycled concrete (RC) aggregates produced from CDW can without issues be utilized to replace concrete aggregates, as they constitute almost seventy percent of the overall volume of concrete. This approach results in a most effective way of saving natural resources, and also saves landfill space and decreases expenses when the expense of the recycling procedure is compared to the cost of extraction of natural resources. The advantages of the usage of RC encompass decreasing the cost of moving CDW to the landfill and moving virgin materials to the construction site. It additionally increases the lifecycle of the landfill via reducing the quantity of waste disposed. At the same token, the realistic utilization of RC in concrete is unusual. Numerous researchers have investigated the benefits of the utilization of concrete with RC partially or wholy replacing natural aggregates (NA). Nováková and Mikulić^18^, concluded that twenty percent replacement of NA with RC aggregate resulted in no negative impact on the strength properties of concrete. On the contrary^18^, it resulted into increase in compressive strength of 5.8% which was accredited to the residual cement contained within the RC. A research performed by Tošić et. al.^19^ determined that incoparating financial, environmental and technical elements, structural concrete containing fifty percent RC would be an optimal proposition.

As the planet apprehends that bioenergy presents a method to decrease CO_2_ emissions, the energy produced by the way of biomass is progressively changing into a more essential method of producing energy. Timber biomass is taken into consideration as a sustainable source of generating electricity and a precious renewable opportunity to replace exhaustible fossil fuels. Nevertheless, a typical byproduct of this activity is wood ash (WA). WA is a leading health hazard and environmental pollutant due to the absence of any emission management policies that are normally costly^20^. The method of managing this waste is either incineration or landfilling. Uncontrolled landfills raise the potential for groundwater contamination from heavy metal leaching in WA and result in airborne diseases to nearby residents. Alternatively, combustion will boost the emission of CO_2_ into the environment. Siddique^21^ revealed that around seventy percent of produced WA is landfilled, which is considered the main reason to explore methods for the utilization of WA in construction materials. Naik^22^ indicated that WA has enormous potential to be utilized as an activator and a pozzolanic mineral admixture in cementitious materials. A compressive strength of 46 MPa was obtained from a mixture resulting from the substitution of twelve percent of cement with WA, thus exceeding the strength of the control mix^23^.

Recently published scholarly work by Beskopylny et al.^24^, experiments conducted on the concrete mix in which rubber tree seed shells replaced coarse aggregates, revealed that the modulus of elasticity, reached a maximum value at a replacement ratio of 4%, while when replacing coarse aggregates with a quantity of 6% or more, a decrease in the modulus of elasticity was detected. Additionally, studies conducted by Mohammed and Sheelan^25^ in which 15% of waste glass powder was utilized as a partial substitute for cement, and (10 and 20%) of crushed waste plastic was utilized as a partial substitute for fine aggregates increased the modulus of elasticity by 6.01% in comparison to reference one. By contrast, replacing sand with plastic for concrete with 15% glass powder led to a decrease of 20% in the modulus of elasticity modulus.

Recently, a comprehensive review of the effects of multiple waste materials replacing normal ingredients in concrete mix design was published^26^. Accordingly, the benefits and shortcomings of including CR, WA, RC, and Waste Glass as waste materials to replace common concrete mix ingredients on the modulus of elasticity of concrete are investigated. The main novelty of this work is that it explores the combination of up to three different types of recycled waste materials on the modulus of elasticity of structural concrete mix design. As a matter of fact, the primary purpose of this project is to accumulate, scrutinize, and assess the impact of those recycled wastes on the produced concrete modulus of elasticity. This mechanical property was selected due to its significance in designing structures for the serviceability limit state, in which the primary emphasis is the control of crack widths and the minimization of deflections. The novelty of this study is the replacement of a combination of three waste materials simultaneously in a single concrete mix.

## Experimental program

### Materials

In this research, the waste components incorporated into the concrete mix are:

#### Wood ash (WA)

**Figure. 1:**
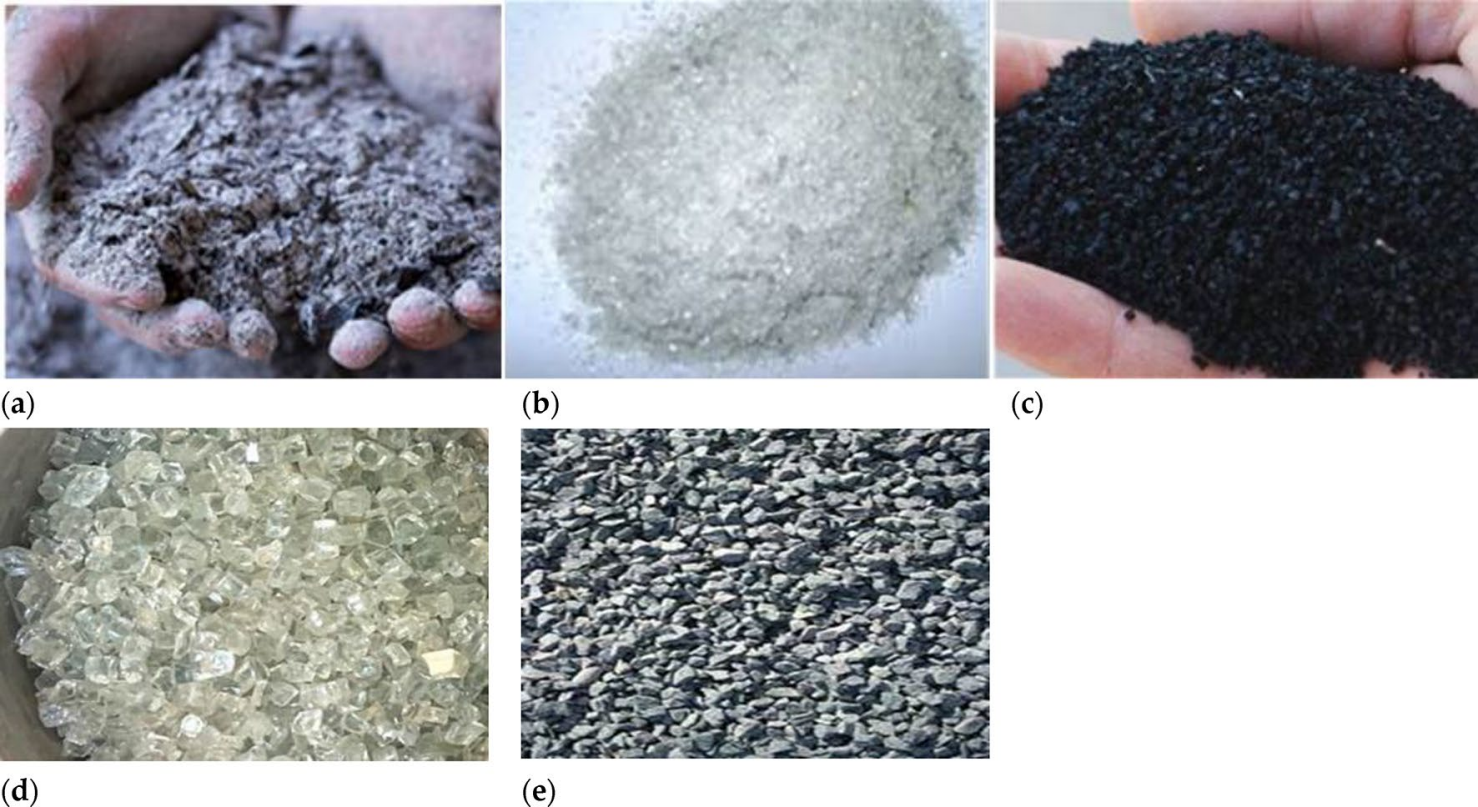
Waste Materials: (**a**) Wood Ash (**b**) Fine Crushed Glass (**c**) Crumb Rubber (**d**) Crushed Glass (**e**) Recycled Concrete.

WA is a byproduct ensuing from the burning of wood in wood bakery (Fig. 1a) with a grain size that varies from 0.13 to 0.60 mm.

#### Fine crushed glass (FCG)

FCG (Fig. 1b) is waste glass from trashed bottles that are crushed and granulated by sieving. The particle size varies from 30 µm down to as fine as 0.1 µm.

#### Crumb rubber (CR)

CR (Fig. 1c) comprises fine rubber particles varying in size from 0.075 mm to no more than 4.75 mm obtained from used tires.

#### Crushed glass (CG)

CG (Fig. 1d) is waste glass from trashed bottles that are crushed on a roller. They consist of particles with sizes ranging from 10 to 40 mm.

#### Recycled concrete (RC)

RC (Fig. 1e) is obtained from the crushing of demolished concrete components. They consist of particles with sizes ranging from 10 to 40 mm.

**Figure. 2:**
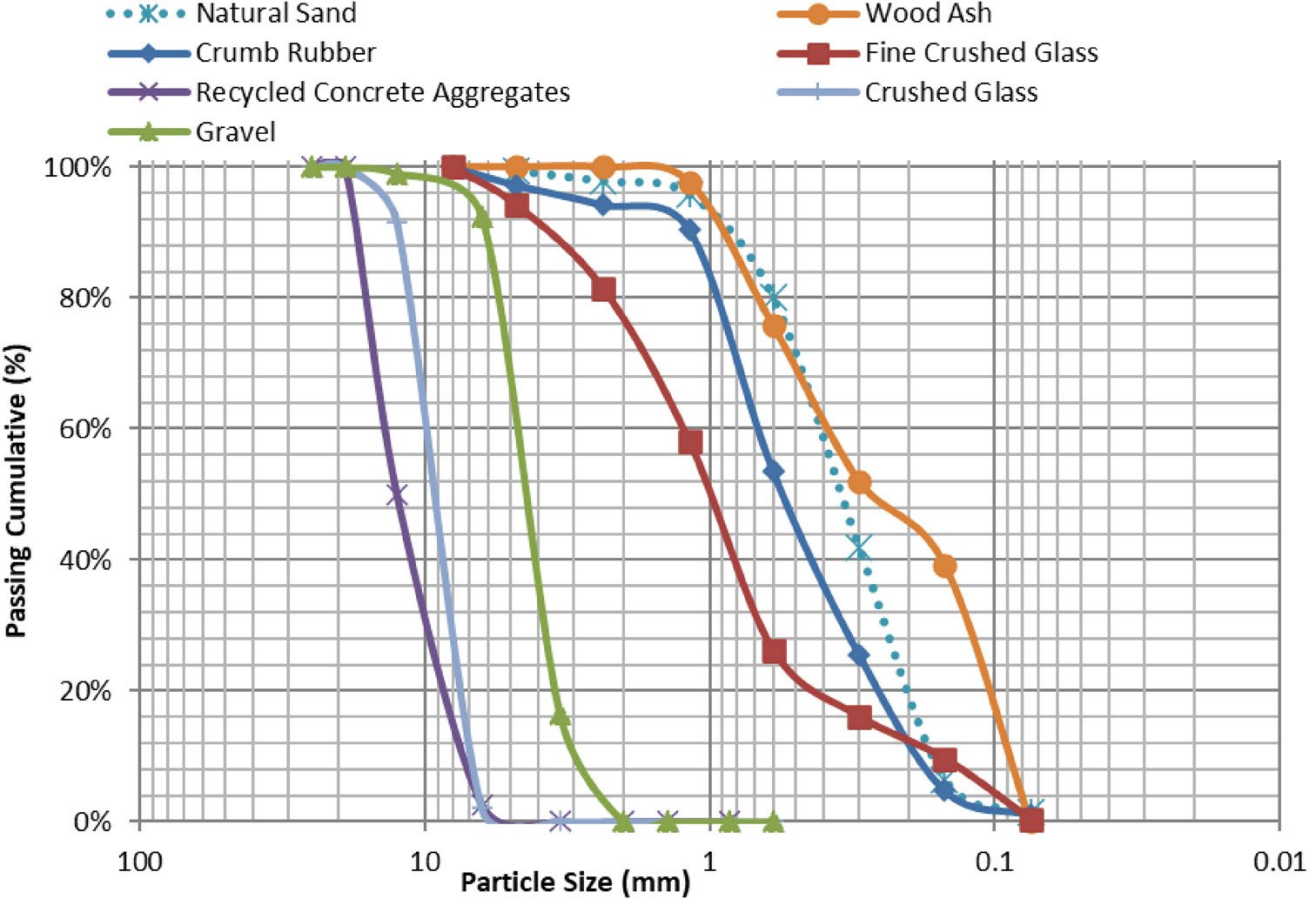
Fine and Coarse Aggregates Materials Sieve Analysis.

ASTM C136^27^ was employed to conduct the sieve analysis for the coarse and fine aggregates. The sieve analysis results for the four fine aggregates and the three coarse aggregates are displayed in Fig. 2. The particle size distribution of CG and RC is similar to that of gravel. The grain size distribution of the four fine aggregates is acceptable and within the limits specified by the ASTM standards.

The chemical compositions of the WA, CR, and CG are listed in Table 1. As for CR, it is the standard composition of rubber tires.

**Table 1:** Chemical Composition of Wood Ash, Crumb Rubber, Crushed Glass, Cement, Sand, and Gravel.

Composition	% WA	% CR	%CG	% Cement	% Sand	% Gravel
SiO_2_	37.86	26.5	72.61	21.20	98.20	12.0
Al_2_O_3_	13.24	8.7	1.38	4.60	0.28	4.5
Fe_2_O_3_	6.91	9.3	0.48	1.80	0.10	3.0
CaO	18.83	12.9	11.70	61.70	0.28	55.8
MgO	4.11	6.4	0.56	4.30	0.03	1.0
Na_2_O	1.39	1.4	13.12		0.03	0.2
K_2_O	2.36	1.1	0.38		0.01	0.2
SO_3_		1.6	0.09	2.00	0.07	
TiO_2_		1.0			0.02	
Ci		0.1				
Zn		20.2				
Loss on ignition	15.3	10.8	0.22	4.40	0.98	23.3

### Concrete mix design

The mix design was performed in a manner to assess the effect of recycled waste materials on the modulus of elasticity (MOE) of the concrete. Several concrete mix design combinations were explored. ASTM C192^28^ was employed for the concrete mixing, casting, curing, and test samples preparation. In this research, a constant water to binder ratio of 0.5 was adopted in the production of concrete mixes. The control mix was a normal mix produced from a combination of 740 kg/m^3^ of sand, 320 kg/m^3^ of cement, 1240 kg/m^3^ of gravel, and 160 kg/m^3^ of water. In excess of sixty-five mix designs were cast by partly replacing cement by WA, natural sand (NS) by CR, and/or FCG, and/or WA, and gravel by CG and/or RC aggregates. Mixes realized by incorporating individual waste materials are displayed in Table 2.

**Table 2:** Individual Waste Type Replacement Percentages.

Waste type	Replaced component	Percentage replacements
WA	Cement	2%, 4%, 6%, 8%, 10%. 15%. and 20%
WA	NS	2%. 10%. 15%. 20%. 25%. and 30%
CR	NS	5%, 10%, 15%, and 10%
FCG	NS	10%, 20%, and 30%
CG	Gravel	5%, 10%, 20%, 30%, 40%, and 50%
RC	Gravel	20%, 25%, 40%, 50%, 60%, 75%, 80%, and 100%

Mixes realized by incorporating two types of wastes are displayed in Table 3.

**Table 3:** Two Waste Types Mix Component Replacement Percentages.

Material	WA: Cement RC: Gravel	WA: Cement CR: NS	WA: Cement CG .Gravel	CR: NS RC : Gravel	CR: NS CG: Gravel	WA: NS RC: Grave/
Percentage	2% WA 25% RC	2% WA 4%CR	4% WA 5% CG	4% CR 25% RC	4%CR 5% CG	10% WA 50% RC
	4% WA 25% RC	4% WA 2% CR	4% WA 10% CG	4% CR 50% RC	4% CR 10% CG	15%WA 75% RC
	2% WA 50% RC	2% WA 2% CR	2% WA 10% CG	2% CR 50% RC	2% CR 10% CG	20% WA 100% RC

Mixes realized by incorporating three types of wastes are displayed in Table 4.

**Table 4:** Three Waste Types Replacement Percentages.

Material	WA: Cement, CR: NS, and RC: Gravel	WA: Cement, CR: NS, and CG; Gravel
Percentages	2% WA + 2% CR + 25% RC	2% WA + 2% CR + 5% CG
	2% WA + 2% CR + 50% RC	2% WA + 2% CR + 10% CG
	2% WA + 4% CR + 25% RC	2% WA + 4% CR + 5% CG
	2% WA + 4% CR + 50% RC	2% WA + 4% CR + 10% CG
	4% W^r^A + 2% CR + 25% RC	4% WA + 2% CR + 5% CG
	4% WA + 4% CR + 25% RC	4% WA + 4% CR + 5% CG
	4% WA + 2% CR + 50% RC	4% WA + 2% CR + 10% CG
	4% WA + 4% CR + 50% RC	4% WA + 4% CR + 10% CG

### Conducted experiments

For each mix, three cylindrical specimens 150 mm by 300 mm were cast and for each experiment the following data were attained according to the applicable ASTM standards:

- 28 days Compressive Strength pursuant to ASTM C39^29^
- MOE pursuant to ASTM C597^30^ and ASTM C469^31^

## Results

In this study, the compressive strength and MOE of more than sixty-five mixes having different percentages of replacement of WA, CR, FCG, CG, and RC aggregates were realized and assessed.

### Compressive strength results

**Figure. 3:**
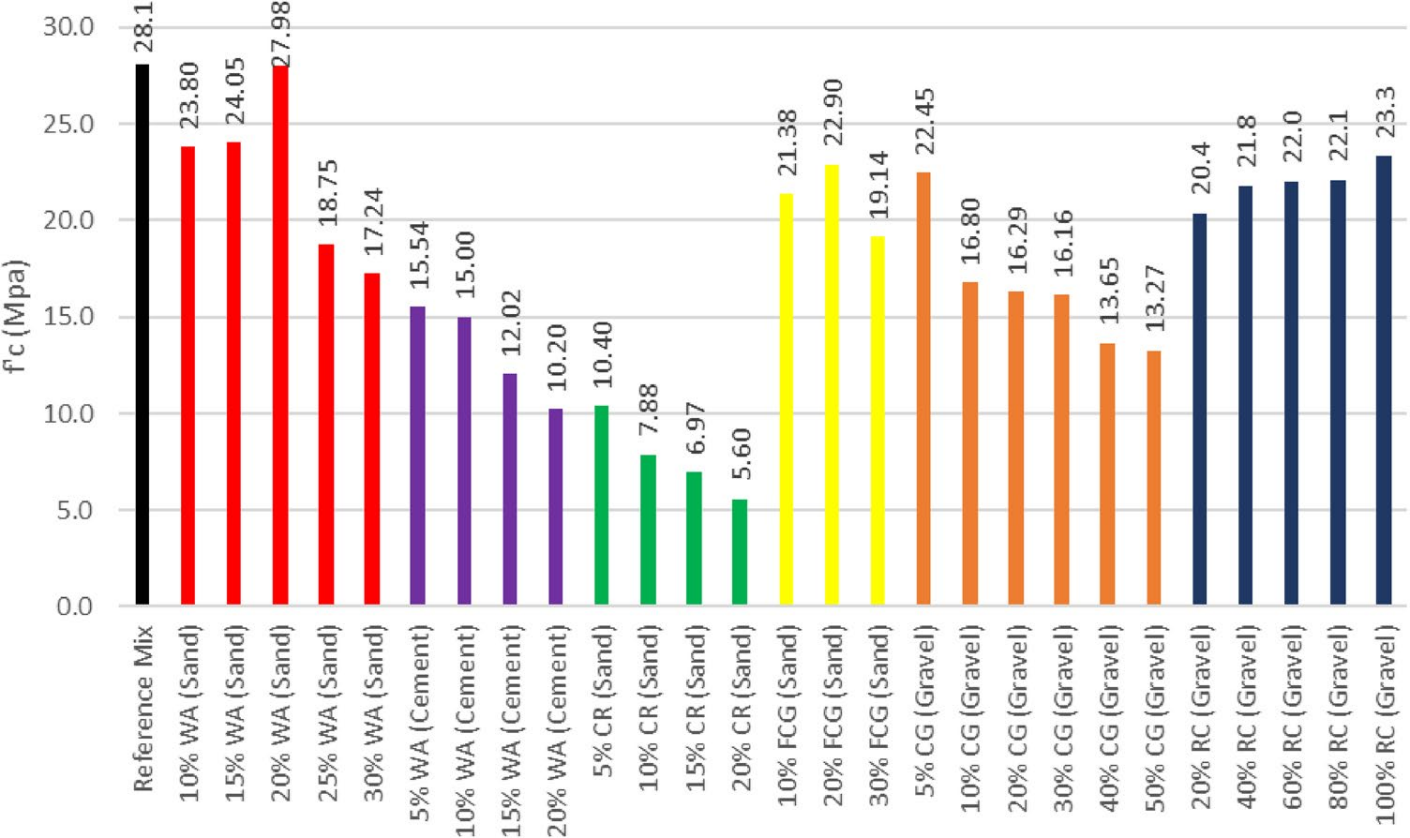
Individual Material Replacement Compressive Strength.

ASTM C39^29^ was employed to obtain the 28-day compressive strength. The results for individual waste materials replacements are displayed in Fig. 3. The compressive strength decreased with the increase of WA replacing cement. The replacement of cement by 5% WA realized the best results. The compressive strength declined with the replacement of 10% of sand by WA but the results displayed a steady increase in the compressive strength up to a replacement ratio of 20%.

Replacing sand with CR resulted in a very sharp decline in the compressive strength. A 50% decline in the compressive strength was obtained with a 5% replacement. To realize an optimum concrete mix, a 2% replacement of sand with CR is adopted. The replacement of sand by FCG at a ratio of 20% achieved an optimal strength. A 40% decrease in strength resulted from the substituting of 10% of the medium aggregates by CG. As for RC replacing gravel, the 40% and below replacement ratio resulted in a strength decrease and a strength increase for above 40%.

**Fig. 4:**
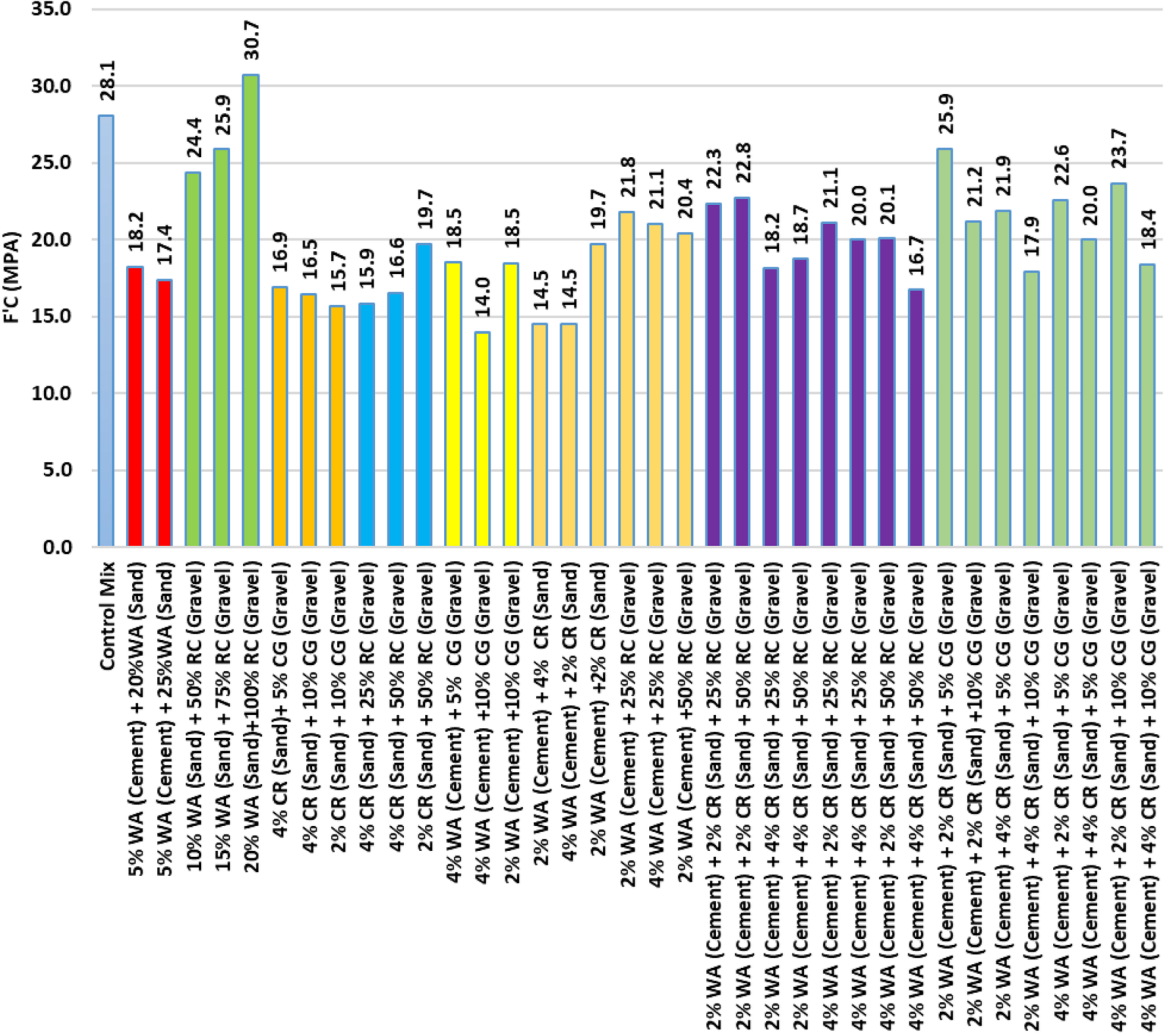
Compressive Strength: Multiple Material Replacement.

The results for two and three waste materials replacements are presented in Fig. 4. The combination of gravel 20% WA replacing sand with 100% RC replacing gravel, and 10% WA replacing sand with 50% RC replacing had exhibited an enhanced compressive strength versus the normal mix. The optimal performance for three waste materials replacements was the combination 2% CR for sand, 2% WA for cement, and 5% CG for gravel yet it was less than the normal mix by 7.83%.

### Modulus of elasticity

**Figure. 5:**
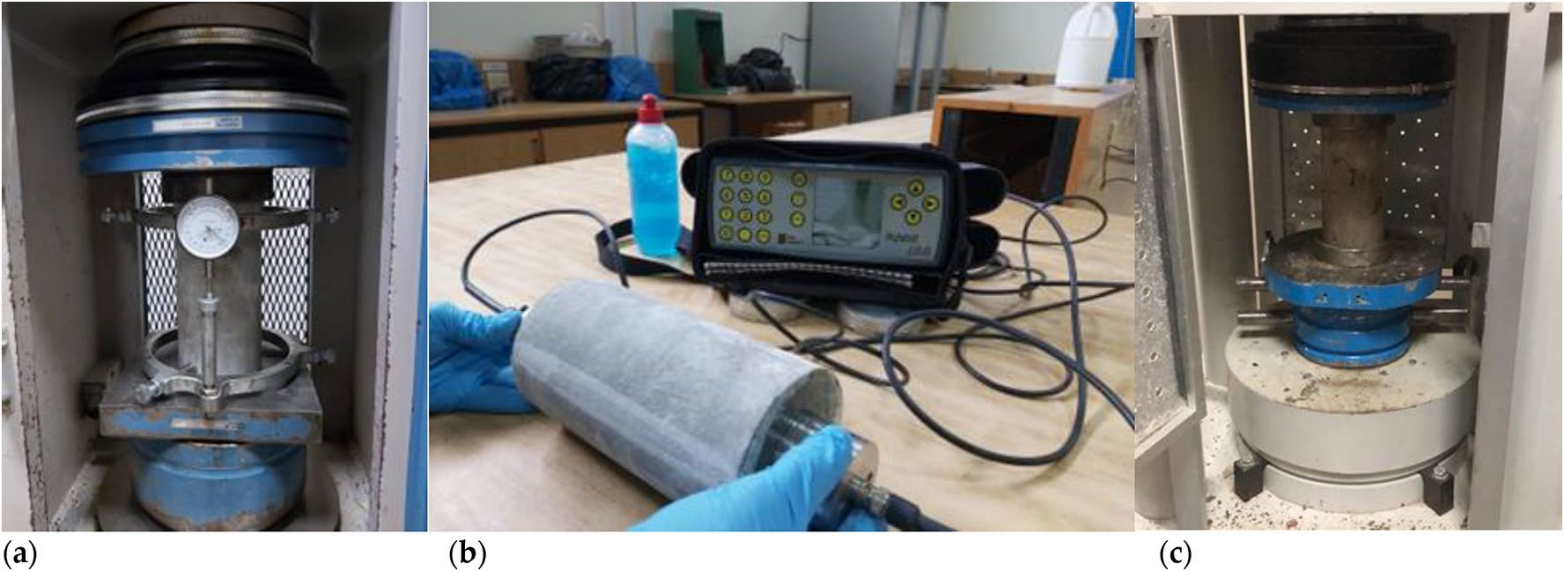
Modulus Elasticity Data Source: (**a**) Compressometer (**b**) Ultra-Sonic Pulse Velocity (**c**) Compressive Strength.

The MOE of concrete (*E*_***c***_***)*** is an essential property that displays its capacity to deform elastically. Building Codes usually necessitate that certain values of MOE must be achieved to guarantee that the structural integrity of the building is adequate and to avert unacceptable distortions. In this study, MOE was determined using three different procedures shown below:

- MOE Test using a Compressometer (Fig. 5a) according to ASTM C469^30^
- MOE Test utilizing Ultra-Sonic Pulse Velocity (Fig. 5b) as specified by ASTM C597^31^
- MOE determined using the 28 days Compressive Strength (Fig. 5c) as specified by ACI 318^32^

**Figure. 6:**
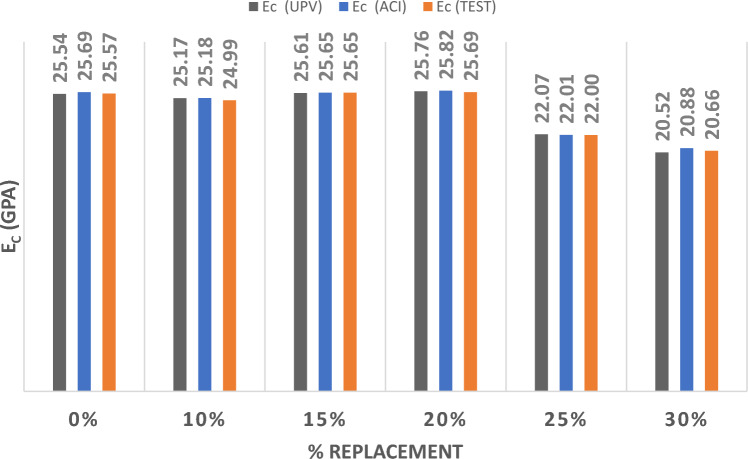
Modulus of Elasticity for Wood Ash Replacing Sand.

Figure 6 represents the results of the three methods utilized to determine if the MOE resulted from partially substituting sand with WA at various percentages. The results show that substituting sand with WA up to 20% would maintain comparable values for the MOE. Nonetheless, the MOE values diminished considerably with 25% and 30% substitution ratios in a magnitude of 12 to 20% decrease in the values of MOE. This could be due to the surge in water absorption due to the increase in WA which caused this drop in MOE.

**Figure. 7:**
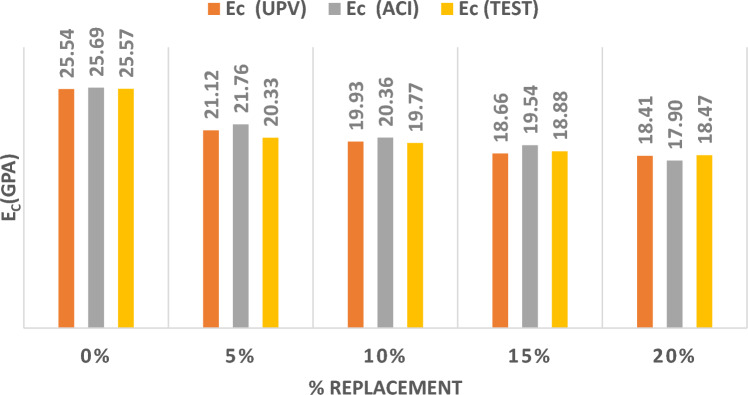
Modulus of Elasticity for WA Replacing Cement.

Figure 7 represents the results of the three methods utilized to attain the MOE resulting from partially replacing cement with WA at various percentages. Replacing cement by WA resulted in a substantial decrease in the MOE versus the control mix at the 5% replacement. This value remained constant as the percentage of replacements increased to reach up to 20%.

**Figure. 8:**
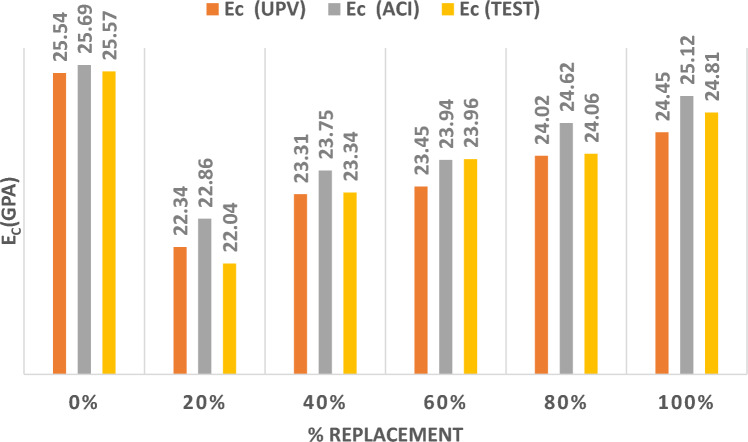
Modulus of Elasticity for RC Replacing Gravel.

Figure 8 represents the outcomes of the three methods utilized to determine the MOE by partially replacing gravel with RC at different percentages. Replacing gravel by RC resulted in the value of the MOE decreasing substantially while comparing a 20% replacement to the control mix and then improved with the increase in the percentage of replacement ratios and becoming almost identical to the results of the control mix at a replacement ratio of 100%.

**Figure. 9:**
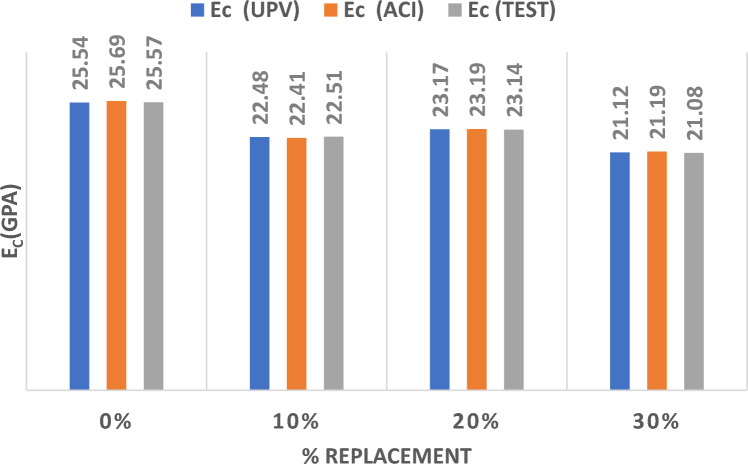
Modulus of Elasticity for FCG Replacing Sand.

Figure 9 represents the results of the three methods utilized to determine the MOE by partially replacing sand with FCG at different percentages. Substituting sand by FCG reduced the value of the MOE by 10% at a substitution ratio of 10% and 20%. A value of 30% substitution resulted in a significant reduction in the value of MOE.

**Figure. 10:**
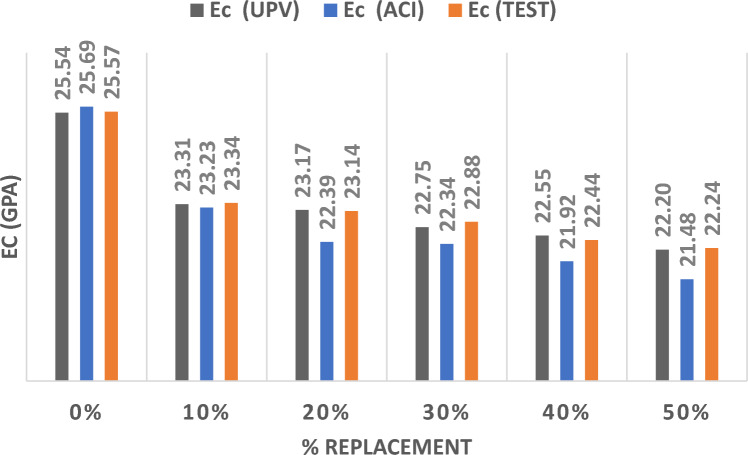
Modulus of Elasticity for CG Replacing Gravel.

Figure 10 represents the results of the three methods utilized to determine MOE by partially substituting gravel with CG at various percentages. Substituting gravel by CG resulted in a significant reduction in the MOE. A reduction of about 20% in the value of MOE was realized as the substitution percentages varied from 5% up to 50%.

**Figure. 11:**
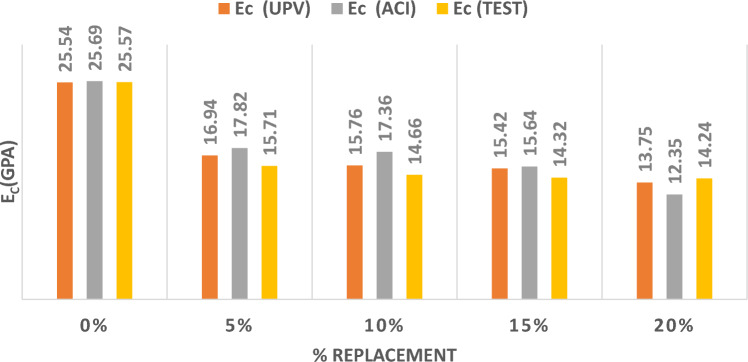
Modulus of Elasticity for CR Replacing Sand.

Figure 11 represents the results of the three methods utilized to determine the MOE by substituting sand with CR at different percentages. Replacing sand by CR has recorded the highest reduction in the MOE with the increase in the replacement values from 5%, 10%, 15%, to 20%. The reduction in the value of the MOE reached up to 50% with a 20% replacement.

Table 5 displays the effect on the modulus of elasticity due to two waste materials replacing natural materials in the concrete mix design. For 5% WA replacing cement and 20% sand, comparable results as the normal mix were obtained, and all three methods do provide the same values. For CR and CG, all the results had values inferior to the normal mix and the methods used were inconsistent. Once again, for CR and RC, all the results had values inferior to the normal mix and the methods used were inconsistent. Finally, 100% RC and 20% WA replacing sand exhibited greater values of MOE compared to the natural mix with the values of each method varying inconsistently.

**Table 5:** Modulus of Elasticity (MPa): Two Waste Materials Replacing Natural Materials.

WA(Cement) + WA(Sand)	Ec (UPV)	Ec (ACI)	Ec (TEST)
0% WA(Cement) + 0%WA(Sand)	25.54	25.69	25.57
5% WA(Cement) + 20%WA(Sand)	25.32	25.67	25.58
5% WA(Cement) + 25%WA(Sand)	21.66	21.72	21.67

WA (sand) + RC (Gravel)	Ec (UPV)	Ec (ACI)	Ec (TEST)
0% WA + 0% RC	25.54	25.69	25.57
10% WA + 50% RC	24.45	25.97	22.37
15% WA + 75% RC	24.96	26.13	24.54
20% WA + 100% RC	27.41	26.28	25.88

CR(sand) + CG(Gravel)	Ec (UPV)	Ec (ACI)	Ec (TEST)
0% CR + 0% CG	25.54	25.69	25.57
4% CR + 5% CG	21.92	21.49	22.01
4% CR + 10% CG	20.92	20.64	20.41
2% CR + 10% CG	20.72	19.22	20.80

CR(sand) + RC(Gravel)	Ec (UPV)	Ec (ACI)	Ec (TEST)
0% CR + 0% RC	25.54	25.69	25.57
4% CR + 25% RC	21.39	20.35	20.73
4% CR + 50% RC	20.33	20.72	20.84
2% CR + 50% RC	23.03	22.91	22.72

WA (sand) + RC (Gravel)	Ec (UPV)	Ec (ACI)	Ec (TEST)
0%WA + 0% RC	25.54	25.69	25.57
10% WA + 50% RC	24.45	25.97	22.37
15% WA + 75% RC	24.96	26.13	24.54
20% WA + 100% RC	27.41	26.28	25.88

Table 6 displays the effect on the modulus of elasticity due to three waste materials replacing natural materials in the concrete mix design. For 2% WA replacing cement, 2% CR replacing sand, and 5% CG replacing gravel, similar results as the normal mix was obtained for all practical purposes. As for the validity of the compatibility of the three methods used, in general, they were consistent with some minor variation for a few mixes.

**Table 6:** Modulus of Elasticity (MPa): Three Waste Materials Replacing Natural Materials.

WA (Cement) + CR (Sand) + RC (Gravel)	Ec (UPV)	Ec (ACI)	Ec (TEST)
0% WA + 0%CR + 0% RC	25.54	25.69	25.57
2% WA + 0%CR + 25% RC	23.31	23.68	23.34
2% WA + 2% CR + 25% RC	23.45	23.20	23.06
2% WA + 4% CR + 25% RC	21.79	21.64	21.01
4% WA + 0%CR + 25% RC	22.82	23.07	23.28
4% WA + 2% CR + 25% RC	23.03	23.11	22.51
4% WA + 4% CR + 25% RC	22.34	22.09	22.02
2% WA + 0%CR + 50% RC	22.41	22.99	22.41
2% WA + 2% CR + 50% RC	23.88	24.30	24.90
2% WA + 4% CR + 50% RC	22.07	21.89	20.40
4% WA + 2% CR + 50% RC	22.62	22.14	22.01
4% WA + 4% CR + 50% RC	21.66	21.29	20.59
2% WA + 2% CR + 0% RC	22.48	22.89	22.01
4% WA + 2% CR + 0% RC	20.33	20.06	20.80
2% WA + 4% CR + 0% RC	19.04	19.82	19.67

WA(Cement) + CR(Sand) + CG(Gravel)	Ec (UPV)	Ec (ACI)	Ec (TEST)
0% WA + 0% CR + 0% CG	25.54	25.69	25.57
2% WA + 2% CR + 5% CG	25.03	25.05	25.14
2% WA + 2% CR + 10% CG	24.88	23.81	23.52
2% WA + 4% CR + 5% CG	22.13	23.21	23.11
2% WA + 4% CR + 10% CG	20.99	21.00	20.40
4% WA + 2% CR + 5% CG	21.32	23.14	23.01
4% WA + 0% CR + 10% CG	21.93	21.59	21.95
4% WA + 2% CR + 10% CG	25.17	24.12	24.52
4% WA + 4% CR + 5% CG	21.05	22.67	22.01
4% WA + 4% CR + 10% CG	20.33	20.04	20.01

## Conclusion

In order to contribute to the overall objective of reducing the emission of CO_2_ due to the widespread utilization of concrete and conserving the natural resources involved in producing concrete, in addition to the advantages of diverting wastes from landfills, this research explored the utilization of WA as a substitution for sand and cement, CR as a substitution for sand, and RC and CG as a substitution for gravel. The CR replacement of sand recorded the lowest moduli of elasticity, which justifies the utilization of only 2% CR. For the replacement of two waste materials, a superior modulus of elasticity was obtained with a 100% RC replacing gravel in combination with a 20% WA replacing sand, achieving an increase of 7.32% for the UPV test and 1.21% for the stress–strain test. As for three waste materials, 2% CR replacing sand, 4% WA replacing sand, and 10% CG replacing gravel provided a comparable result to the control mix. For all three methods, the obtained moduli of elasticity by partially replacing waste with natural materials displayed negligible variations in the results except for mixes incorporating CR.

## Data Availability

All generated data is presented in the paper.
